# An unusual hematopoietic stem cell transplantation for donor acute lymphoblastic leukemia: a case report

**DOI:** 10.1186/s12885-020-6681-2

**Published:** 2020-03-06

**Authors:** Di Zhou, Ting Xie, Suning Chen, Yipeng Ling, Yueyi Xu, Bing Chen, Jian Ouyang, Yonggong Yang

**Affiliations:** 1 Department of Hematology, The Affiliated Drum Tower Hospital of Nanjing University Medical School, 321 Zhongshan Road, Nanjing, 210008 People’s Republic of China; 2 Jiangsu Institute of Hematology, First Affiliated Hospital of Soochow University, Suzhou, People’s Republic of China

**Keywords:** Donor leukemia, Acute lymphoblastic leukemia, Hematopoietic stem cell transplantation, SF3B1, BRAF

## Abstract

**Background:**

Donor acute lymphoblastic leukemia with recipient intact is a rare condition. We report a case of donor developing acute lymphoblastic leukemia 8 yrs after donating both bone marrow and peripheral blood hematopoietic stem cells.

**Case presentation:**

This case report describes a 51-year old female diagnosed with acute lymphoblastic leukemia who donated both bone marrow and peripheral blood stem cells 8 yrs ago for her brother with severe aplastic anemia. Whole exome sequencing revealed leukemic genetic lesions (SF3B1 and BRAF mutation) only appeared in the donor sister, not the recipient, and an unusual type of hematopoietic stem cell transplantation with the recipient’s peripheral blood stem cells was done. The patient remained in remission for 3 months before disease relapsed. CD19 CAR-T therapy followed by HLA-identical unrelated hematopoietic stem cell transplantation was applied and the patient remains in remission for 7 months till now.

**Conclusions:**

This donor leukemia report supports the hypothesis that genetic lesions happen randomly in leukemogenesis. SF3B1 combined with BRAF mutation might contribute to the development of acute lymphoblastic leukemia.

## Background

Donor leukemia is a rare condition and only anecdotal reports described its development [1, 2]. There are two types of donor leukemia, literally the donor itself developing leukemia or donor cells in the recipient developing leukemia (Donor Cell Leukemia, DCL), which is more commonly reported. Two mechanisms have been proposed for DCL development: occult transfer of malignant cells from donors during hematopoietic stem cell transplantation (HSCT) or leukemic transformation of healthy donor cells in recipients [3–6]. The latter one is unpredictable but it takes up the majority of DCL. Donor developing leukemia with recipient intact is even a rarer condition and only few reports described the development of acute myeloid leukemia (AML), not acute lymphoblastic leukemia (ALL), in peripheral blood stem cell (PBSC) donors [7, 8]. G-CSF administration was suspected for leukemogenesis but there have been no valid evidence to support the hypothesis. Meanwhile, there have been no reports describing the development of ALL in hematopoietic stem cell donors, not recipients.

Here we reported a case with only the donor developing ALL 8 yrs after donating hematopoietic stem cells but not the recipient. Whole exome sequencing (WES) showed leukemic genetic lesions (SF3B1 and BRAF mutation) only appeared in the donor’s hematopoietic cells. A special type of HSCT using the recipient’s PBSCs was done. To our knowledge, this is the first case report of donor developing ALL and an unusual type of HSCT. We hope it could provide more evidence for donor leukemia formation and the potential role of SF3B1 and BRAF mutation in ALL.

## Case presentation

**Fig. 1:**
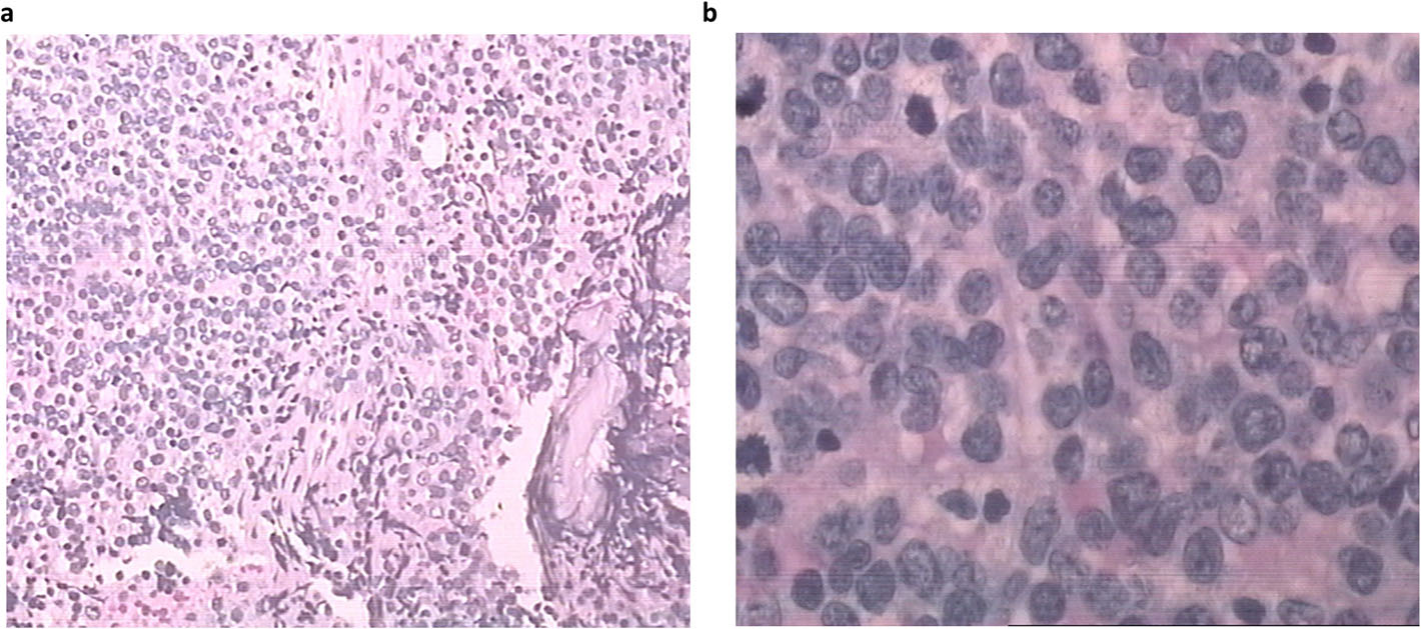
Bone marrow biopsy of the patient showed a markedly hypercellular marrow implying ALL

In May 2017, a 51-year old female presented to our hospital with over 1 week of fatigue. She has medical history of donating both bone marrow and PBSCs 8 yrs ago for her brother with severe aplastic anemia (SAA). G-CSF was used to mobilize hematopoietic stem cells for PBSC collection. Complete blood count (CBC) showed lymphocytosis (Lymphocyte 10*10^9/L), moderate anemia (Hb 61 g/L) and thrombocytopenia (PLT 20*10^9/L). Peripheral blood smear demonstrated 30% of blast cells. Bone marrow biopsy showed lymphoblastic leukemia involving a markedly hypercellular marrow (Fig. 1). Flow cytometry showed the blasts expressed CD10, CD19, CD22, CD38, HLA-DR, but not CD13, CD33, CD117, or cytoplasmic MPO. Chromosomal analysis showed a normal female karyotype. The diagnosis of B-ALL was made. VDP regimen (Vindesine, Daunorubicin, Prednisone) was started after her diagnosis and bone marrow aspirate 14 days later revealed complete remission (CR). Then 2 cycles of VDLP regimen (Vindesine, Daunorubicin, Prednisone, Pegaspargase) and 2 cycles of MA (Mitoxantrone, Cytosine arabinoside) + Pegaspargase (PEG-Asp) regimen were administered. During this period, the patient remained in remission and cerebrospinal fluid (CSF) remained clear after intermittent four time lumbar punctures. Minimal residue disease (MRD) monitoring by flow cytometry after each cycle did not detect blast cells with abnormal phenotype. Considering she has no other HLA-identical siblings or unrelated donors from China Bone Marrow Bank at that time and her brother, who was perfectly stable, a special type of auto-HSCT using her brother’s PBSCs was performed in December 2017. Before the transplantation, whole exome sequencing (WES) was done for both the donor and recipient to rule out occult genetic abnormalities in the stem cells. Results showed that only the patient, not her brother, has genetic mutations including SF3B1 and BRAF mutation associated with hematological malignancies in her hematopoietic cells, not oral mucosal cells (Table 1) [9–11]. Myeloablative conditioning regimen (Melphalan 140 mg/m^2^ d-3/d-2/d-1, Cytarabine 1 g/m^2^ d-3/d-2, Cyclophosphamide 60 mg/Kg d-3/d-2) was used. A total of 2.34*10^6/kg CD34+ cells, 13.0*10^8/kg MNCs from peripheral blood were collected and transfused into the patient. Neutrophil engraftment occurred at day + 11 and platelet engraftment occurred at day + 14. VP regimen (Vindesine, Prednisone) and MTX + 6-MP regimen were used for maintenance therapy. Bone marrow aspirate and flow cytometry showed that the patient remained in complete remission 3 months after transplantation. In May 2018, the patient’s CBC showed leukocytosis and bone marrow aspirate indicated disease relapsed with 53.33% blast cells. Flow cytometry showed the blasts expressed CD10, CD19, HLA-DR and partially expressed CD11b. Genetic testing using polymerase chain reaction (PCR) technique found that SF3B1 and BRAF exome mutation was negative. Re-induction chemotherapy with VDP regimen was administered, the patient achieved CR. However, after another 2 cycles of high-dose MTX+ PEG-Asp regimen, disease relapsed. In November 2018, CD19 CAR-T therapy followed by HLA-identical unrelated hematopoietic stem cell transplantation was applied and the patient remains in remission for 7 months till now.

**Table 1:** Genetic mutations in the sister’s hematopoietic cells (Sample A), oral mucosal cells (Sample B) and the brother’s hematopoietic cells (Sample C) detected by WES

	Gene	Chrs	Position	Ref Allele	Alt Allele	Predicted Protein Variants
Genetic mutations shared by Sample A,B,C	**ARHGEF10L**	**1**	**18,014,104**	**G**	**A**	**V794M, V972M, V719M, V1016M, V977M**
	**YIF1B**	**19**	**38,798,111**	**T**	**C**	**Y234C, Y232C, Y249C, Y246C, Y218C**
	**AMBRA1**	**11**	**46,529,825**	**C**	**T**	**R662H, R755H, R633H**
	**ATRAID**	**2**	**27,438,205**	**T**	**C**	**C80R, C22R, C135R**
	**CACNB3**	**12**	**49,212,737**	**G**	**A**	**G9R**
	**IPO4**	**14**	**24,650,793**	**G**	**C**	**L991V**
	**PLA2G15**	**16**	**68,293,133**	**C**	**T**	**P271L**
	**ZNF574**	**19**	**42,582,813**	**T**	**C**	**S109P, S19P**
	**NID2**	**14**	**52,505,486**	**T**	**G**	**N746H**
	**ALG8**	**11**	**77,824,984**	**C**	**T**	**G242E**
	**HOXB5**	**17**	**46,670,899**	**T**	**C**	**Y49C**
	**TIE1**	**1**	**43,787,288**	**T**	**G**	**Y1083D, Y1038D**
	**CACNA1F**	**X**	**49,066,102**	**G**	**C**	**S1603C, S1614C, S1549C**
	**FREM2**	**13**	**39,263,181**	**C**	**G**	**T567R**
Genetic mutations shared by Sample A and C	**ZGRF1**	**4**	**113,506,711**	**C**	**T**	**E1305K, E1363K**
	**SLC43A2**	**17**	**1,495,007**	**C**	**T**	
	**CHM**	**X**	**85,134,073–85,134,073**	**–**	**A**	
Genetic mutations specific to Sample A	**SF3B1**	**2**	**198,267,483**	**C**	**T**	**R625H**
	**BRAF**	**7**	**140,481,411**	**C**	**T**	**G466E**
	**ZC3H7B**	**22**	**41,742,094**	**G**	**C**	**R516P**
	**LAMC1**	**1**	**183,077,450**	**C**	**T**	**R255C**

Sample A: the sister’s hematopoietic cells; Sample B: the sister’s oral mucosal cells; Sample C: the brother’s hematopoietic cells

## Discussion and conclusion

Leukemogenesis is a complex process involving accumulation of genetic changes in self-renewing hematopoietic stem cells (HSCs) [12]. Two-hit model was proposed for the clonal evolution of leukemia [13]. Genome sequencing indicated that most mutations in the leukemia genome were random events and the acquirement of one or two initiating mutations generated the founding clone for leukemogenesis [14]. In this case, a careful review of the medical history of both the brother and sister showed that there were no noteworthy differences in hazard substance exposure. During HSCT, the brother was exposed to more cytotoxic drugs, including CTX, MTX, CsA, while the sister was only exposed to short-time G-CSF before she developed ALL. WES revealed leukemic alterations including SF3B1 and BRAF mutation only happened in the donor sister. So we assumed that development of these mutations in her hematopoietic stem cells, not oral mucosal cells, were probably random events.

SF3B1 gene is located at chromosome 2q33.1 and the encoded protein is an essential component of the U2 small nuclear ribonucleoproteins complex (U2 snRNP), which functions by splicing pre-mRNAs. SF3B1 mutation often occurs in melanoma, chronic lymphocytic leukemia (CLL) or myelodysplastic syndrome (MDS) [15]. Quesada et al. found that SF3B1 mutation could possibly alter its normal function by changing the physical interactions with its binding partners [16]. Of note, U2 snRNP with mutated SF3B1 could affects the splicing of FOXP1 pre-mRNA resulting in the expression of truncated FOXP1 protein, which has been implicated in diffuse large B-cell lymphoma [17]. In this case, WES found SF3B1 R625H mutation in the patient’s blood cells but there have not been reports about the association between SF3B1 mutation and ALL. Considering the mutation could affect genes involved in malignant transformation of B cells like FOXP1, in might participate in the patient’s leukemogenesis.

BRAF gene is located at chromosome 7q34 and it encodes a member of the Raf kinase family which regulates cell division, differentiation and secretion. More than 30 mutations of BRAF gene have been identified in human cancers and V600E was most frequently found [18]. It is a likely driver mutation in hairy cell leukemia and has been widely observed in melanoma, Langerhans cell histiocytosis, papillary thyroid carcinoma, colorectal cancer and non-small-cell lung cancer [19]. BRAF mutations could change the activation segment from inactive state into active state and enhance B-raf kinase activity, which augment cell proliferation. However, in ALL the frequency of BRAF mutation is very low and only V600E, G468A, L597Q mutations have been reported [20–22]. Helene Cav et al. reported that children with Noonan Syndrome, some of which harbor BRAF mutation, are at higher risk of developing ALL [23]. In this case, we found BRAF G466E in the patient, not her brother. It might form double hits with SF3B1 mutation, which could affect genes involved in pre-B cell differentiation, and ultimately leads to leukemogenesis in this patient. More basic research needs to be done to clarify the mechanism.

*ZC3H7B* and *LAMC1* gene mutations were also found in the patient’s hematopoietic cells, but there have been no reports of them in hematological malignancies. Their roles in leukemogenesis still need to be uncovered.

G-CSF is used in the majority of allogeneic hematopoietic stem cell donations. Some studies showed its application was associated with increased risk of later development of MDS/AML [6, 8]. Meanwhile, some other studies failed to identify the correlation [24, 25]. It is a critical issue but it remains controversial. In our case, the donor developed ALL 8 yrs after hematopoietic stem cell mobilization using G-CSF. Her leukemogenesis and G-CSF usage were probably irrelevant since G-CSF is a regulator for granulopoiesis, not lymphopoiesis. The duration and dosage of G-CSF administration was longer and higher in the recipient brother, but till now no signs of MDS/AML have been detected in him. Therefore, high quality and more conclusive clinical data is needed to clarify G-CSF usage and leukemia formation especially in healthy donors.

The suspicion of delayed onset of DCL was also considered. According to previous reports of DCL, the average latent period is within 4 yrs of HSCT [26, 27]. In our case, the recipient brother did not develop leukemia 8 yrs after transplantation. Additionally, WES showed no leukemia-associated gene mutations in him and it further ruled out the possibility of delayed leukemia onset.

Auto-HSCT can be considered in MRD negative ALL patients without appropriate HLA-identical donors. In this case, a special type of auto-HSCT was performed. Compared with stem cells collected from the patient herself, peripheral blood stem cells from her brother were leukemia cell free and it was supposed to lower the risk of disease relapse. However, the patient relapsed 3 months after transplantation. Since SF3B1 and BRAF mutation turned negative after disease relapsing, we hypothesized that leukemia sub-clones outgrew and caused disease relapse.

To our knowledge, this is the first case report with only donor developing ALL but not the recipient and this is also the first report of an unusual type of auto-HSCT. We hope it could provide more evidence for the double-hit model in leukemogenesis and a potential alternative for donor leukemia treatment.

## Data Availability

The clinical data used or analyzed in this case report are available from the corresponding author on reasonable request.

## Abbreviations

ALL: Acute Lymphoblastic Leukemia
Allo-HSCT: Allogenic Hematopoietic Stem Cell Transplantation
AML: Acute Myeloid Leukemia
ATG: Anti-Thymocyte Globulin
Auto-HSCT: Autologous Hematopoietic Stem Cell Transplantation
CsA: Cyclosporine A
CSF: Cerebrospinal Fluid
CTX: Cyclophosphamide
DCL: Donor Cell Leukemia
G-CSF: Granulocyte-Colony Stimulating Factor
GVHD: Graft Versus Host Disease
HSC: Hematopoietic Stem Cell
MNC: Mononuclear Cell
MTX: Methotrexate
PBSC: Peripheral Blood Stem Cell
PCR: polymerase chain reaction
SAA: Severe Aplastic Anemia
U2 snRNP: U2 small nuclear ribonucleoproteins complex
WES: Whole Exome Sequencing
